# Optimized Centrifugation and Activation Protocol for the Preparation of Plasma Rich in Growth Factors in Pigs

**DOI:** 10.3390/biomedicines14030640

**Published:** 2026-03-12

**Authors:** Michela Maria Taiana, Andrea Massimiliano Nebuloni, Elena De Vecchi, Laura de Girolamo, Giuseppe Michele Peretti, Enrico Ragni, Arianna Barbara Lovati

**Affiliations:** 1 Laboratorio di Biotecnologie Applicate all’Ortopedia, IRCCS Ospedale Galeazzi-Sant’Ambrogio, Via Cristina Belgioioso 173, 20157 Milan, Italyandreamassimiliano.nebuloni@grupposandonato.it (A.M.N.);; 2 Laboratory of Clinical Chemistry and Microbiology, IRCCS Ospedale Galeazzi-Sant’Ambrogio, Via Cristina Belgioioso 173, 20157 Milan, Italy; 3 Department of Biomedical Sciences for Health, University of Milan, Via Mangiagalli 31, 20133 Milan, Italy; 4 Équipe Universitaria di Ortopedia Rigenerativa e Ricostruttiva, IRCCS Ospedale Galeazzi-Sant’Ambrogio, Via Cristina Belgioioso 173, 20157 Milan, Italy; 5 Cell and Tissue Engineering Laboratory, IRCCS Ospedale Galeazzi-Sant’Ambrogio, Via Cristina Belgioioso 173, 20157 Milan, Italy; arianna.lovati@grupposandonato.it

**Keywords:** musculoskeletal disease, regenerative medicine, platelet-rich plasma, plasma rich in growth factors, porcine model, osteoarthritis, translational medicine, cartilage

## Abstract

**Background:** Cartilage defects remain a clinical challenge due to the limited intrinsic repair capacity of hyaline cartilage, driving increasing interest in blood-derived products, including platelet-rich plasma (PRP). Variability in PRP preparation and activation protocols limits reproducibility and clinical translation, particularly in large animal models where species-specific differences are an additional cue. This study aimed to standardize and optimize in pigs a protocol for plasma rich in growth factors (PRGF), a leukocyte-poor PRP, aligned with current human clinical practice. **Methods:** Whole blood from six female pigs was processed via three centrifugation protocols and activated with varying CaCl_2_ concentrations to evaluate gelation and morphology. PRGF was characterized through hematological analysis, ELISA-based quantification of soluble factors, and structural imaging of fibrin gel via histology and scanning electron microscopy. Data were further analyzed using protein–protein interaction networks, hierarchical clustering, and comparative human PRGF proteomic profiles. **Results:** Protocol with 400× *g* centrifugation followed by 13.3 mM CaCl_2_ activation achieved the most favorable performance, yielding the highest platelet recovery, effective leukocyte clearance, and consistent formation of a well-organized fibrin network. Porcine activated PRGF showed substantial overlap in detected factors and concentration ranges with human activated PRGF prepared with the same protocol. **Conclusions:** These findings establish a robust, clinically aligned porcine PRGF protocol and support the pig as a relevant translational model for PRP-based regenerative strategies, providing a reliable platform for preclinical evaluation of cartilage therapies.

## 1. Introduction

Cartilage defects remain a significant clinical challenge because adult hyaline cartilage possesses limited intrinsic reparative capacity. This limitation has driven increasing interest in regenerative medicine and biological augmentation strategies such as autologous platelet derivatives and bioactive matrices to improve both the quality and persistence of cartilage repair [1]. Platelet-rich plasma (PRP) has emerged as a widely used orthobiologic, owing to its reservoir of platelet-derived growth factors, such as, among others, platelet-derived growth factors (PDGFs), transforming growth factor beta 1 (TGFβ1), vascular endothelial growth factor (VEGF), and insulin-like growth factor (IGF), and its ability to modulate inflammation and enhance tissue repair [2,3,4]. However, the clinical performance of PRP is hampered by substantial heterogeneity in preparation methods, which may affect platelet enrichment, leukocyte content, and activation protocols [2,5]. In this frame, the classification system proposed by Dohan Ehrenfest et al. distinguishes between leukocyte-poor PRP (LP-PRP) and leukocyte-rich PRP (LR-PRP), recognizing their divergent biological profiles [6]. Leukocyte-rich formulations can promote the release of pro-inflammatory cytokines, potentially exacerbating synovial irritation in chondral lesions, whereas leukocyte-poor systems, such as those that aim to deliver a cleaner, anti-inflammatory, and regenerative product [5,7]. Additionally, the activation of platelets leads to further release of bioactive molecules [7]. Even if activation may naturally occur when platelets get in contact with tissue collagen via an FcRγ-chain-mediated mechanism, in clinical practice, this is generally obtained by thrombin or calcium chloride supplementation after a standardized low centrifugation step leading to minimal leukocyte contamination, all features associated with high reproducibility and clinical safety [8,9]. As a consequence of activation, a fibrin gel embedded with the regenerative molecules is also formed, which may be used on wounds and tears for contact-driven regeneration of tissues [10], including cartilage [11].

For translational relevance of research studies aimed at implementing regenerative approaches, large animal models are essential, although differences with humans in terms of blood properties and regenerative capacities emerged [12]. In this scenario, the porcine model is particularly advantageous due to its joint size, cartilage thickness, and biomechanical properties closely resembling the human knee [13,14,15]. Although several porcine studies have explored PRP in wound healing, ligament repair, and experimental osteoarthritis [16,17,18,19], rigorous standardization of PRP protocols in pigs remains limited, especially regarding growth factor composition, leukocyte depletion, and gel features. Porcine blood also differs in platelet reactivity and hematologic composition from human blood, underscoring the need for species-specific optimization rather than a direct adaptation of clinical human protocols that may affect reliability [13].

Given this expanding interest in PRP-based therapies in animal models for an effective clinical translation of in vivo results into human clinical practice, there is a need to establish reliable and reproducible porcine PRP protocols to achieve consistent platelet enrichment and leukocyte depletion, produce fibrin gels with predictable polymerization behavior, and characterize growth factor and cytokine profiles. In this context, the adoption of specialized pipelines, such as the protocol for the preparation of plasma rich in growth factors (PRGF) that belongs to the family of leukocyte poor platelet enriched products (LP-PRP) already validated in humans [20] and other species [21,22], may be a valuable starting point to substantially reduce variability, although species-specific optimization may still be required to achieve optimal outcomes. In this perspective, previous data in the porcine model gave valuable results about efficacy, although a thorough optimization of the PRGF protocol was not performed [23].

The present study was designed to address the current lack of standardization in porcine blood-derived products. The primary objective was the identification of the optimal centrifugation and activation protocol for PRGF preparation in pigs, maximizing platelet enrichment while minimizing leukocyte contamination and ensuring reproducible fibrin gel formation. Additionally, this work addressed several secondary objectives, including the characterization of the soluble factor profile released from activated PRGF, the analysis of donor-dependent variability, and the correlation between platelet counts and released biomolecules. Finally, a comparison between porcine PRGF and human PRGF prepared with the same protocol was performed to validate the translational potential of this large-animal model. By explicitly defining these parameters, this study provides a standardized framework to improve the consistency and predictability of PRGF applications.

## 2. Materials and Methods

### 2.1. Animals and Blood Collection

Six female pigs (Large White, mean age: 6.67 ± 0.52 months; mean body weight: 82.17 ± 4.49 kg) were included in this study. Blood was collected from pigs enrolled in an acute non-recovery study authorized by the Italian Ministry of Health (Authorization No. 771/2020-PR, issued on 30 July 2020). Therefore, in accordance with the 3Rs principle of animal research, specifically to reduce the total number of animals used, an *a priori* power analysis was not conducted. This logistical integration ensured maximal data utility from the subjects while adhering to ethical institutional protocols. Peripheral blood was collected under general anesthesia from the femoral vein of each animal into tubes containing 3.2% sodium citrate as an anticoagulant.

### 2.2. Blood Processing

Approximately 50 mL of whole blood (WB) samples were processed using the Endoret^®^ system V VET (BTI Biotechnology Institute, Vitoria, Spain) according to three centrifugation protocols: P1, 580× *g* for 8 min at room temperature (RT); P2, 400× *g* for 8 min at RT; and P3, 300× *g* for 8 min at RT. Following centrifugation, plasma was separated into two components: the 50% top portion containing platelet-poor plasma or fraction 1 (F1) and the 50% bottom portion containing platelet-rich plasma or fraction 2 (PRGF, F2). Then, 500 µL samples were positioned in wells in a 48-well plate and supplemented with three different concentrations of stock 10% calcium chloride dihydrate solution (CaCl_2_ ∗ 2H_2_O) (final 0.1%—6.7 mM, 0.2%—13.3 mM, or 0.4%—26.6 mM) to induce the activation at 37 °C in a Plasmaterm H device (BTI Biotechnology Institute). Gelation was assessed at 30 min and 60 min post-activation. Gel formation was first evaluated qualitatively on a subjective scale, scoring the visual absence or presence of either unstable or stable and either transparent or opaque polymerized gel in the well. Further, after positioning the formed gel, if possible, on the cover of the plate, the size of the gel was assessed with a ruler on a 3 mm increase scale, namely, up to 3 mm, 6 mm, or 9 mm. This allowed us to define three main classes: 0–3, from absence of gel to unstable and transparent gel of 9 mm size; 4–7, different types of stable but semi-transparent gel from 3 mm to 9 mm in size; 8–10, different types of stable and opaque gel from 3 to 9 mm in size. Gel formation was evaluated by three independent observers who were blinded to the experimental protocols. To minimize interobserver variability, observers were pre-trained by an external coordinator to categorize gel appearance based on predefined criteria (presence/absence of polymerization, stability, and opacity). Size determination was performed using a standard ruler. For qualitative scoring, consensus was reached through discussion to ensure data integrity.

### 2.3. Hematological Analyses

Complete counts on whole blood and PRGF samples were performed using an automated hematology analyzer (Sysmex XN-2000 hemocytometer, Sysmex Italia S.r.l., Milano, Italy).

### 2.4. Soluble Factor Quantification by ELISA

Before quantification, samples were kept frozen at −80 °C until use. Then, 250 µL was twofold diluted before secreted factors detection with the enzyme-linked immunosorbent assay (ELISA) Quantibody^®^ Porcine Cytokine Array Q50 (RayBiotech, Peachtree Corners, GA, USA) following the manufacturer’s protocol and four technical replicates. Concentrations were determined by comparison with standard samples, taking into account sample dilution. Values are reported as pg/mL for those factors always detected across all samples. The following factors were tested: IL4, IL6, IL8, IL10, IL12p40p70, GM-CSF, IFNγ, TGFβ1, TNFα, CCL3L1, IFNα, IL1α, IL1ra, IL13, IL17A, IL18, MIG, MIP1b, PECAM1, Decorin, GASP1, IGFBP5, IL15, IL-22, Insulin, IP10, MCP1, NCAM1, TWEAK R, ANG1, IL17F, MIF, OPG, PDGF-BB, RANTES, TGFα, TIMP1, TIMP2, VEGF, Eotaxin-1, EPO, FGF21, Galectin-9, IFNβ, IGF2, IL21, IL28B, PlGF2 and SCF.

### 2.5. Protein–Protein Interaction Network Generation

Interactome maps of ELISA-identified proteins were generated with the STRING tool http://www.string-db.org (accessed on 17 December 2025) (database v12.0) [24]. The following settings were selected: (i) organism: *Sus scrofa*; (ii) network type: full STRING network with the edges indicating both functional and physical protein associations; (iii) meaning of network edges: evidence with line color indicating the type of interaction evidence; (iv) sources for active interaction evidence: text mining, experiments, databases, co-expression, neighborhood, gene fusion and co-occurrence; (v) minimum required interaction scores: medium confidence (0.400, default value). This score does not reflect the strength or the specificity of the interaction; rather, it represents a confidence measure, indicating how likely STRING considers the interaction to be true based on the available evidence. All scores rank from 0 to 1, with 1 being the highest possible confidence. A score of 0.5 would indicate that roughly every second interaction might be erroneous (e.g., a false positive).

### 2.6. Histology

Platelet gels were fixed overnight in 10% neutral-buffered formalin at 4 °C, rinsed in distilled water, and then transferred to 70% ethanol until processing. Pellets were manually embedded in paraffin following standard dehydration and clearing steps: graded isopropanol (90%, 100%), xylene, and paraffin infiltration at 60 °C. Embedded samples were allowed to solidify on a −5 °C cooling plate and sectioned at 7 µm using a rotary microtome (Leica Biosystems, Deer Park, IL, USA). Sections were floated on a 50 °C water bath, collected on Superfrost/Plus slides (VWR International Srl, Milano, Italy) and dried overnight at 37 °C. For H&E staining, slides were deparaffinized in xylene, rehydrated through graded ethanol (100%, 90%, 70%), and rinsed in distilled water. Nuclei were stained with Mayer’s hematoxylin for 5 min, blued under running tap water (or Scott’s substitute) for 5 min, and counterstained with eosin Y for 5 min. Slides were dehydrated in ethanol, cleared in xylene, mounted with an appropriate medium, and dried under a chemical hood. Images were acquired with an Olympus BX43 microscope (Olympus, Tokyo, Japan) and processed with cellSens Imaging Software v4.4.1 (Olympus).

### 2.7. Scanning Electron Microscopy

Platelet gels were fixed in 0.1 M sodium cacodylate buffer (pH 7.2) containing 2.5% glutaraldehyde and 3.7% formaldehyde for 1 h at RT. Following fixation, samples were rinsed in ultrapure distilled water for 30 min and subsequently dehydrated through a graded ethanol series (25%, 50%, 70%, 80%, 90%, 95%, and 100%). Dehydrated specimens were then incubated in increasing ratios of ethanol to hexamethyldisilazane (2:1, 1:1, and 1:2; 15 min each) and finally transferred to 100% hexamethyldisilazane overnight until complete evaporation. Dried samples were mounted onto aluminum stubs, sputter-coated with platinum (Sputter ACE600, Leica Microsystems, Mannheim, Germany), and imaged using a FESEM Sigma field-emission scanning electron microscope (Zeiss, Oberkochen, Germany) operated at 5 kV.

### 2.8. Hierarchical Clustering

Hierarchical clustering was obtained with the ClustVis package https://biit.cs.ut.ee/clustvis/ (beta version, accessed on 15 December 2025) [25]. For hematological values, data were ln(x+1) transformed. No row scaling was applied. Both rows and columns were clustered using correlation distance and average linkage. Other parameters were: (i) clustering distance for rows and columns, correlation; (ii) clustering method for rows and columns, average; and (iii) tree ordering for rows and columns, tightest cluster first.

### 2.9. Human PRGF ELISA-Data Retrieval

Soluble factors in activated human PRGF supernatants, obtained with the same category of device and P2 protocol, were retrieved from a previous publication [26].

### 2.10. Statistical Analyses

Data were expressed as mean ± standard deviation (SD). For the analysis of soluble factors and hematological values, potential outliers were sifted with the ROUT method (Q set at 1%), and identified values were removed. Further, a Shapiro–Wilk normality test was conducted (significance alpha level set at 0.01). With normally distributed values, an RM one-way ANOVA followed by a Tukey post hoc test was performed if there were no missing values. Otherwise, a mixed-effects model followed by a Tukey post hoc test was preferred. Significance was set at *p* ≤ 0.05. For correlation analysis, with at least one non-normally distributed data point in the matrix, an exploratory Spearman r correlation coefficient was calculated (two-tailed, 95% confidence interval) without correction for multiple comparisons.

## 3. Results

### 3.1. Performance of PRGF Protocols

The blood values of freshly collected WB were within the normal range of values for Large White pigs (Table 1A).

**Figure 1:**
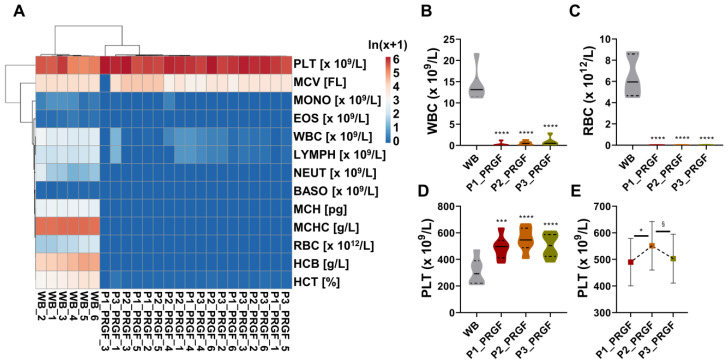
Hematological characterization of whole blood (WB) and plasma rich in growth factors (PRGF) obtained using three centrifugation protocols. (**A**) Hierarchical clustering analysis showing a clear segregation between WB and PRGF samples across all protocols based on the ln(x+1) transformed blood values, with the sample clustering tree shown at the top. The color scale indicates absolute amount: red shades = high amount and blue shades = low amount. (**B**) Leukocyte clearance of residual white blood cells (WBC) in PRGF relative to WB. (**C**) Red blood cell (RBC) depletion in PRGF compared with WB. (**D**) Platelet (PLT) concentration in PRGF relative to WB. (**E**) Relative PLT enrichment (fold increase PRGF/WB) in PRGF samples. (**B**) through (**E**): data are reported as mean ± SD, n = 6. § for *p*-value ≤ 0.1; * for *p* ≤ 0.05; *** for *p* ≤ 0.001; **** for *p* ≤ 0.0001.

After processing, hierarchical clustering showed a sharp dichotomy between WB and PRGF samples obtained with all protocols (Figure 1A). WBC clearance, evaluated as the percentage of residual leukocytes in PRGF compared to WB, was highest in P1 (2% ± 4), followed by P2 (4% ± 4) and P3 (7% ± 7) (Table 1B and Figure 1B). Regarding RBC, the highest depletion occurred for P1 and P2 (0.3% ± 0.1 vs. WBC), followed by P3 (0.5% ± 0.3) (Table 1B and Figure 1C). Eventually, all protocols led to a significant PLT increase with respect to WB (308.7 × 10^9^/L ± 92.3). The mean platelet concentration was highest in P2, with a value of 551.0 × 10^9^/L ± 83.3, followed by P3 (502.8 × 10^9^/L ± 84.0) and P1 (489.7 × 10^9^/L ± 81.4) (Table 1B and Figure 1D). The relative platelet increase (fold increase PRGFP/WB) confirmed the superiority of P2, with a mean value of 1.9 ± 0.5, compared to 1.7 ± 0.4 for both P1 and P3. Due to the overall similar performances of the three centrifugation protocols, the PLT values were directly compared with P2, resulting in significantly different values from P1 and P3 (Figure 1E). This was further corroborated by correlation analysis of PLT levels in PRGFs vs. WB, where P2 had the lower Pearson r value (0.491) with respect to P1 (0.607) and P3 (0.626), although statistical significance was not reached. Comparing only PRGFs, P3 had a lower r value of 0.813 (*p*-value 0.049) vs. P1 and of 0.778 (*p*-value 0.068) vs. P2, with respect to P1 and P2, which had the highest and most significant similarity (r of 0.933, *p*-value 0.007). Overall, P3 showed the least effective performance, while P1 and P2 gave similar outcomes, with P2 being endorsed with the highest PLT concentration.

### 3.2. Gelation Time and Gel Quality

**Figure 2:**
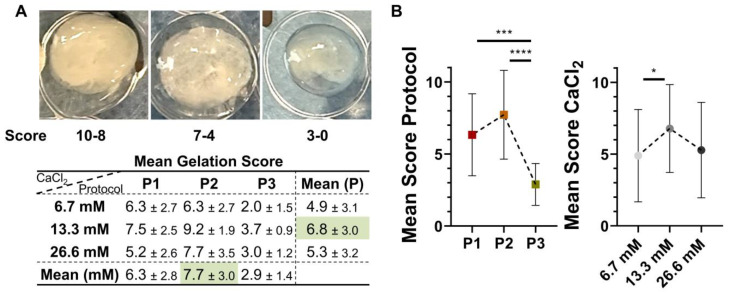
Semi-quantitative scoring of platelet gel formation under different protocols and calcium concentrations. (**A**) Representative macroscopic images illustrating the gelation score used for the evaluation (from 0, no gelation, to 10, maximal gelation, given by a subjective evaluation of both stable or unstable and transparent or opaque appearance, coupled with size of up to 3, 6 or 9 mm, see M&M), together with a summary table reporting gelation scores across centrifugation protocols (P1–P3) and CaCl_2_ concentrations. (**B**) Graphical representation of the gelation scores shown in panel (**A**), plotted by protocol and by CaCl_2_ concentration. Data are reported as mean ± SD, n = 6. * for *p* ≤ 0.05; *** for *p* ≤ 0.001; **** for *p* ≤ 0.0001.

Gelation occurred within approximately 30 min at 37 °C across all protocols, and was completed at 60 min (Figure 2A). P2 protocol gave rise to the most stable gels, followed by P1, while P3 often gave rise to incomplete polymerization (Figure 2B). Also, CaCl_2_ concentration affected gel formation and resulted in more effective gel formation at 13.3 mM across all protocols (Figure 2B), although in a context of higher similarity across conditions.

**Figure 3:**
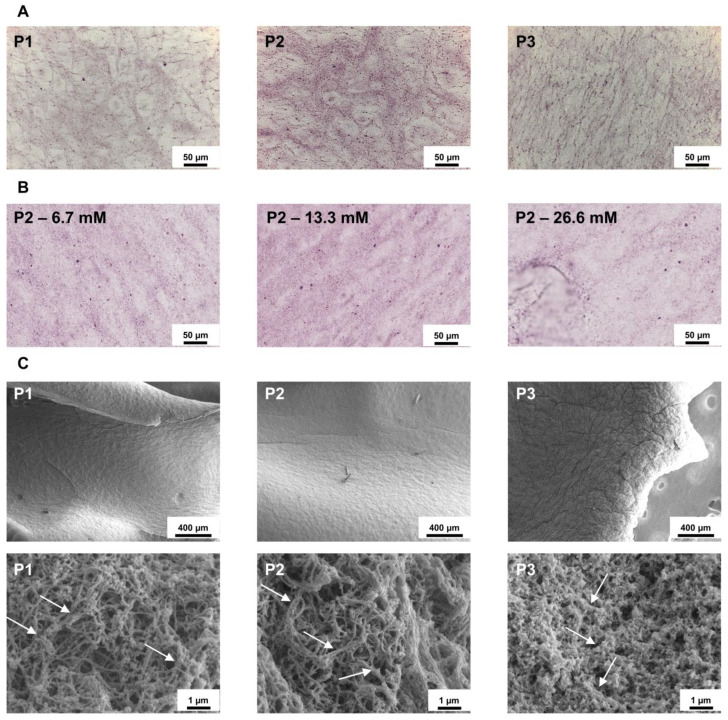
Histological and ultrastructural characterization of platelet-derived gels obtained with different centrifugation protocols and CaCl_2_ concentrations. (**A**) Representative hematoxylin/eosin–stained sections showing fibrin-rich, proteinaceous matrices in gels generated with protocols P1–P3. P1 and P2 display prominent eosinophilic (pink) fibrin matrix, with P2 exhibiting the most intense and homogeneous staining, whereas P3 shows weak eosin staining consistent with limited gelation and poor matrix formation. 40× images are shown. Note that individual fibril morphology is below the resolution limit of light microscopy. (**B**) Representative hematoxylin/eosin staining of gels activated with different CaCl_2_ concentrations, showing comparable fibrin staining across all conditions. 40× images are shown. Note that individual fibril morphology is below the resolution limit of light microscopy. (**C**) Representative scanning electron microscopy images illustrating protocol-dependent differences in gel ultrastructure: at low magnification (150×), P1 and P2 exhibit a smooth and organized surface, while P3 shows a rough, wrinkled and less organized morphology; at higher magnification (40,000×) of the inner gel region, well-defined fibrin fibrils are evident in P1 and P2, appearing longer and thicker in P2, whereas P3 displays a poorly organized, amorphous structure. Arrows indicate representative large strands of well-organized (P1 and P2) or amorphous fibrin fibrils.

The results were further corroborated by histological analysis. Hematoxylin-eosin staining revealed that P1 and P2 generated a dense fibrin-rich, proteinaceous matrix, evidenced by prominent eosinophilic (pink) fibrous structures (Figure 3A). Among these, P2 showed the most intense and homogeneous eosinophilic signal, indicating a more compact and uniformly organized fibrin network. In contrast, P3 displayed only weak eosin staining, consistent with limited gelation and poor matrix formation. Concerning CaCl_2_, all three concentrations showed similar staining (Figure 3B). Consistent with these findings and focusing on protocol performance, scanning electron microscopy highlighted protocol-dependent differences in gel ultrastructure. At low magnification, P1 and P2 exhibited a comparable, smooth surface morphology, whereas P3 showed a less organized surface characterized by increased roughness and wrinkling (Figure 3C). At higher magnification of the inner gel portion, P1 and P2, mirroring the hematoxylin/eosin results, revealed well-defined fibrin fibrils, longer and thicker in P2, while P3 displayed a poorly organized, more amorphous structure (Figure 3C).

### 3.3. Analysis of Soluble Factors in Activated PRGF

**Figure 4:**
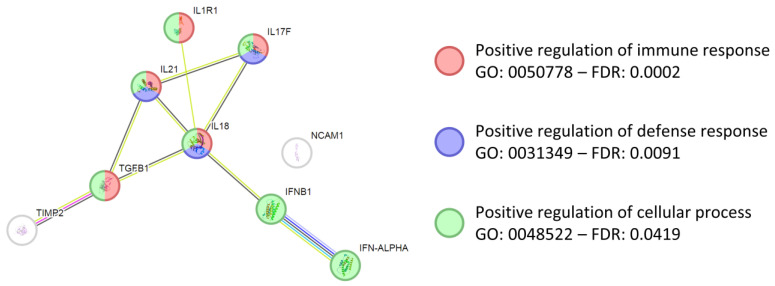
Functional association network for the ≥10 ng/mL proteins in activated PRGF. Connection colors: light blue for curated databases, purple for experimentally determined, green for gene neighborhood, red for gene fusions, blue for gene co-occurrence, yellow for gene neighborhood, black for gene fusions, and purple for gene co-occurrence. Empty nodes, proteins of unknown three-dimensional (3D) structure; filled nodes, known or predicted 3D structure. Regenerative-associated GO terms with FDR are shown.

Based on data previously reported, the analysis of soluble factors was performed across the three protocols in the supernatants of PRGF activated with 13.3 mM CaCl_2_ using a porcine-specific kit (Quantibody^®^ Porcine Cytokine Array Q50, RayBiotech, Peachtree Corners, GA, USA). Out of 50 tested proteins, 34 were present in all samples (Table 2). IFNα had the highest concentration, always close to 100 ng/mL, followed by IL21 and TGFβ1. In the ≥10 ng/mL group, IL17/18/1RA, IFNβ, NCAM1 and TIMP2 were detected. Interestingly, a gene ontology (GO) search highlighted the involvement of these most abundant proteins in several terms related with immune and defense responses and activation of cellular processes, including those positive and crucial for tissue regeneration such as positive regulation of immune response (GO: 0050778), positive regulation of defense response (GO: 0031349) and positive regulation of cellular process (GO: 0048522) (Figure 4).

**Figure 5:**
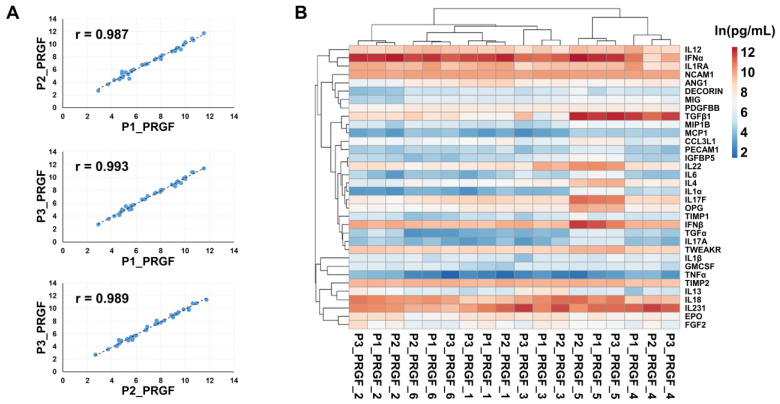
Correlation analysis and hierarchical clustering of activated samples obtained with different protocols. (**A**) Spearman correlation analysis showing a strong concordance among the three protocols, with correlation coefficients (r) consistently ≥0.985 (*p* ≤ 0.0001). In each graph, dots represent one of the 34 detected proteins, ln(pg/mL) transformed. (**B**) Hierarchical clustering of individual activated samples demonstrates predominant grouping by donor identity rather than by protocol, highlighting a strong donor-dependent effect.

Correlation analysis revealed a strong concordance among the three protocols, with Spearman coefficients consistently ≥0.985 (*p* ≤ 0.0001) (Figure 5A). When all samples were analyzed individually, hierarchical clustering showed that activated samples primarily grouped according to donor identity, indicating a predominant donor effect rather than segregation by protocol (Figure 5B). Within this framework, two main clusters emerged, comprising subjects 1/2/3/6 and subjects 4/5. This clustering was mainly driven by higher levels of specific factors in subject 5, namely TGFβ1, IFNβ, IL17F, IL22, OPG, IL4, IGFBP5, IL6, IL1α, TGFα, and IL17A, and by elevated TGFβ1 levels in subject 4. MIP1B was also increased in subjects 4 and 5, although its differential abundance was less pronounced. To further investigate potential protocol-dependent effects at the single-molecule level, each factor was analyzed individually across samples. No significant differences among protocols were detected, except for IL17A, which showed a trend toward reduced levels in P3 compared with both P1 and P2 (*p* ≤ 0.1).

**Figure 6:**
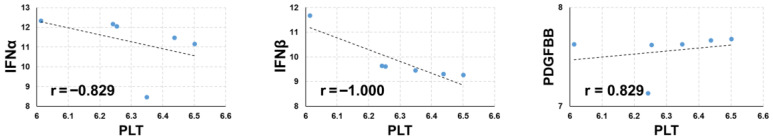
Significant correlations in activated P2_PRGF between platelet counts before activation and soluble factors after activation. Spearman correlation analysis identified three strong (Spearman r ≥ 0.8) and statistically significant (*p* ≤ 0.05) associations. Platelet levels positively correlated with PDGFBB, consistent with platelet activation, while negative correlations were observed with the inflammation-related factors IFNα and IFNβ. For each parameter, the ln(x) transformed data are presented.

To get rid of the donor effect, a correlation analysis was conducted on the most performant protocol (P2) coupled with the most effective CaCl_2_ concentration (13.3 mM). Considering the soluble factors, several players showed a high degree of correlation (Spearman r ≥ 0.8 or ≤−0.8, *p* ≤ 0.05) (Table 3A), being positive for the majority of them. Eventually, considering platelet number before CaCl_2_ supplementation, three strong (Spearman r ≥ 0.8) and significant (*p* ≤ 0.05) correlations emerged (Figure 6 and Table 3B). Confirming platelet activation, PDGFBB had a positive Spearman r (0.829). On the contrary, inflammation-related IFNα and β had a negative one (−0.829 and −1). Widening the analysis on those factors having a significant *p* ≤ 0.05 regardless of the correlation strength, the other 6 factors were identified with negative correlation values (Table 3B).

### 3.4. Comparison Between Porcine and Human Activated PRGFs

The factors detected in porcine activated PRGF produced with P2 and 13.3 mM CaCl_2_ activation were compared with those identified with an identical ELISA technology, in its human release covering a larger number of proteins but belonging to the same functional categories, in human activated PRGF obtained with the same protocol and device, and previously reported [25]. Of note, when shared by porcine and human ELISA arrays, 22 out of 22 pig proteins were also detected in human samples, confirming that the porcine model may efficiently resemble the human scenario (Supplementary Table S1). The analysis of the most abundant porcine factors (≥10 ng/mL group) that were also detected in humans gave similar results, namely TGFβ1 (pig 41 ng/mL vs. human 59 ng/mL), TIMP2 (pig 13 vs. human 23), and IL17F (pig 12 vs. human 10). Of note, pro-inflammatory mediators that may alter regenerative processes were always detected at low levels (≤1 ng/mL) with comparable amounts, namely IL1β (pig 261 pg/mL vs. human 932), IL6 (pig 98 vs. human 28), IL17A (pig 81 vs. human 225), and TNFα (pig 14 vs. human 121). Eventually, two protective factors were more present in porcine than in human activated PRGF, namely IL4 (pig 2168 vs. 528) and especially IL13 (pig 921 vs. human 42).

## 4. Discussion

In this study, we identified and standardized a porcine PRGF preparation protocol that achieves optimal platelet enrichment, effective leukocyte depletion, reproducible calcium-induced fibrin gel formation, and, after activation, a growth factor profile closely aligning with clinically used human PRGF. This will strengthen the translational relevance of the porcine model for regenerative cartilage therapies.

Porcine PRP was prepared with a device configured for veterinary use and activated with a calcium-based protocol routinely used in clinical practice. Although all tested protocols generated PRGF products distinct from whole blood, clinically meaningful differences emerged. In evaluating these protocols, all analyzed parameters, including cellular enrichment, leukocyte depletion, and fibrin gel properties, were weighted equally to identify the most balanced preparation for translational use. Accordingly, protocol P2 (centrifugation at 400× *g* for 8 min), the one identical to current clinical practice in humans, achieved the most favorable balance between platelet enrichment (2×) and erythrocyte/leukocyte depletion, reproducing LP-PRP formulations commonly used for regenerative applications in humans [27]. Of note, the obtained number of platelets (approximately 600 × 10^9^/L) was lower than the concentration generally considered effective in human PRP (1000 × 10^9^/L), although the European Society for Sports Traumatology, Knee Surgery and Arthroscopy Orthobiologics Initiative (ESSKA-ORBIT) consensus did not support a clear correlation between platelet numbers and clinical response [1], taking also into account the role of platelet activity over their mere concentration [27]. Also, this value may be species-specific and therefore related to both porcine donors and the whole blood platelet level. In a previous study to obtain platelet concentrate in pigs, a similar enrichment fold (2.8×) was reported that, starting from a higher basal level (400 vs. 300 × 10^9^/L), was able to allow a final platelet concentration close to 1000 × 10^9^/L [23]. Platelet activation with CaCl_2_ resulted in predictable gelation kinetics across all protocols. However, gel quality and structural integrity differed substantially. P2 consistently produced homogeneous and stable fibrin gels with organized fibrillar architecture, resembling clinically used matrices [28]. The identification of 13.3 mM CaCl_2_ as the optimal activation condition further supports standardization and reproducibility in both experimental and clinical settings, being the standard value used in clinical practice.

Consistent with clinical observations in humans, donor-related variability emerged as the dominant source of molecular heterogeneity in PRGF, outweighing protocol-dependent differences once activation conditions were standardized. Indeed, the clustering of samples primarily according to donor rather than preparation protocol suggests that intrinsic biological factors may play a more relevant role than minor procedural variations in shaping the final molecular composition of the product. This observation closely mirrors what has been reported in clinical applications of platelet-derived products, where inter-individual variability in platelet content, growth factor release, and cytokine profiles can significantly influence therapeutic performance. From a translational perspective, these findings highlight the importance of considering donor-specific biological variability when interpreting experimental outcomes and when designing standardized preparation pipelines. Overall, this finding further strengthens the translational value of the porcine model, as it reproduces one of the major challenges encountered in clinical applications [29,30]. Exploratory correlation analyses confirmed a strong association between platelet content and platelet-derived growth factor B (PDGFBB) release, as previously reported [31,32], together with an inverse correlation with interferon-related inflammatory mediators, supporting a platelet-driven, regenerative-oriented biological output. Consistently, IFN was associated with a severe impairment of platelet aggregation [33]. However, these associations may also reflect donor-specific immunological differences rather than a direct mechanistic effect of the preparation protocol.

Beyond technical performance, the direct comparison between activated porcine and human PRGFs obtained with the identical technology provides a key translational validation in orthopedics. When shared in the ELISA arrays for both species, 22 proteins in porcine activated PRGF were also identified in human samples, according to previous work from our group [26]. While species-specific kits were necessarily employed to ensure cross-reactivity and analytical accuracy for each model, both studies utilized identical suppliers and the same multiplex technological platform. Of note, this consistency across analytical methods greatly reduces the potential impact of technical variability on quantitative comparisons, demonstrating a remarkable qualitative overlap between species. Although absolute concentrations may be influenced by species-specific antibody affinities, the use of a standardized platform allows for a more reliable assessment of the relative biomolecule profiles. Importantly, the most abundant factors in porcine PRGF were present at comparable concentrations, including TGFβ1 [34], TIMP2 [35], and IL17F [36]. These proteins are known to play central roles, both protective and destructive, in tissue homeostasis, extracellular matrix regulation, and cartilage turnover. This quantitative similarity strongly supports the biological equivalence of the optimized porcine PRGF to its human counterpart.

Equally relevant from a clinical perspective in orthopedic regenerative medicine, pro-inflammatory cytokines, such as IL1β [37], IL6 [38], IL17A [39], and TNFα [40], were consistently detected at low concentrations in both species. This low-inflammatory profile aligns with the therapeutic rationale of PRGF in degenerative joint diseases, where minimizing inflammatory burden is critical to promote tissue repair [41]. Interestingly, activated porcine PRGF showed higher levels of anti-inflammatory and protective cytokines, particularly IL4 [42] and IL13 [43], compared with human PRGF. While this difference should be taken into account when interpreting in vivo outcomes, it may also represent a favorable biological window in the porcine model, potentially enhancing tissue protection without deviating from the overall human-like molecular signature.

From a clinical standpoint, these data underscore, particularly when it comes to preclinical studies intended for translation, the importance of selecting and validating PRP protocols in preclinical models that are not only biologically effective but also procedurally identical to those used in patient care. The demonstration that porcine PRGF prepared with P2 and 13.3 mM CaCl_2_ closely reproduces the human PRGF protein profile significantly enhances the predictive value of the porcine model for clinical translation. This alignment reduces the risk of misleading preclinical results driven by species- or protocol-specific artifacts, and supports the use of pigs as a robust model for evaluating PRGF-based strategies before clinical implementation.

This study has some limitations. The sample size was limited, potentially reducing sensitivity to detect minor protocol-dependent differences, especially in the context of a strong donor effect that mirrors clinical variability. Consequently, the study may have been underpowered to reach statistical significance for certain parameters showing non-significant trends, such as the observed tendency for reduced IL-17A levels in P3. Indeed, being a technical study, this was not powered to detect subtle protocol-dependent differences in individual soluble mediators. Furthermore, the strong donor-dependent clustering observed in the cytokine analysis highlights a degree of biological variability that likely necessitates larger cohorts in future studies to fully delineate individual factor kinetics. The analysis focused on technical performance, fibrin architecture, and soluble factor profiles, without direct functional testing on target cells or in vivo efficacy, which will be required to fully link protocol optimization to biological outcomes. Crucially, the present work focuses on technical and analytical standardization. As such, it does not evaluate in vivo cartilage regeneration or disease modification. Further functional and preclinical studies will therefore be necessary to assess the actual biological and therapeutic effects of this optimized PRGF preparation. Regarding the characterization of the fibrin matrix, future work would benefit from more objective measurements of physical gel properties, such as clot mass, volume, or automated image-based quantification of fibrin density to complement the current descriptive analysis. Introducing functional assays at this stage would have confounded protocol-driven effects with cell-specific responses. Platelet activation was performed using calcium chloride only, but alternative activation mechanisms were not explored. The pig–human comparison was restricted to analytical overlap and concentration ranges, without addressing species-specific differences in tissue responsiveness. Finally, this study was not designed to compare different devices or preparation systems, but rather to replicate and standardize a clinically established PRGF preparation pipeline in a porcine model, thereby prioritizing translational alignment over broad methodological generalizability.

## 5. Conclusions

This study identifies a porcine PRGF preparation protocol that is technically robust, biologically consistent, and fully aligned with current human clinical practice. The strong qualitative and quantitative overlap between porcine and human PRGF profiles validates the porcine model as a highly relevant translational system for PRP-based therapies. By reinforcing continuity across preclinical and clinical settings, these findings provide a solid framework for the development, optimization, and clinical validation of PRGF therapies.

## Figures and Tables

**Table 1:** Hematological values of the analyzed samples compared to standard ranges for White Large pigs.

		(A)	(B)		
	Range	WB	P1_PRGF	P2_PRGF	P3_PRGF
**WBC (×10^9^/L)**	11–22	14.09 ± 3.50	0.26 ± 0.44	0.51 ± 0.45	0.88 ± 0.94
**RBC (×10^12^/L)**	5–7	6.40 ± 1.69	0.02 ± 0.01	0.02 ± 0.01	0.03 ± 0.01
**HGB** **(g/L)**	100–140	112.50 ± 27.45	0.00 ± 0.00	0.00 ± 0.00	0.00 ± 0.00
**HCT** **(%)**	30–40	39.15 ± 9.50	0.08 ± 0.04	0.12 ± 0.04	0.15 ± 0.08
**MCV** **(FL)**	50–70	61.50 ± 2.66	52.77 ± 36.54	63.88 ± 26.21	48.88 ± 7.87
**MCH** **(pg)**	15–20	17.65 ± 0.85	0.00 ± 0.00	0.00 ± 0.00	0.00 ± 0.00
**MCHC** **(g/L)**	300–350	287.33 ± 4.07	0.00 ± 0.00	0.00 ± 0.00	0.00 ± 0.00
**PLT (×10^9^/L)**	200–400	308.67 ± 92.29	489.67 ± 81.42	551 ± 83.30	502.83 ± 84.04
**NEUT (×10^9^/L)**	4–10	5.15 ± 3.50	0.00 ± 0.00	0.01 ± 0.01	0.022 ± 0.02
**LYMPH (×10^9^/L)**	6–12	7.86 ± 0.61	0.23 ± 0.44	0.35 ± 0.47	0.83 ± 0.90
**MONO (×10^9^/L)**	0.5–1.5	0.76 ± 0.35	0.01 ± 0.01	0.12 ± 0.25	0.03 ± 0.03
**EOS (×10^9^/L)**	0.2–1	0.23 ± 0.08	0.00 ± 0.00	0.01 ± 0.01	0.01 ± 0.01
**BASO (×10^9^/L)**	0–0.2	0.10 ± 0.03	0.00 ± 0.00	0.00 ± 0.00	0.00 ± 0.00

(**A**) for whole blood samples. (**B**) for PRGF samples. Mean values ± SD, n = 6.

**Table 2:** Soluble factors in activated PRGFs.

(pg/mL)	P1_PRGF	P2_PRGF	P3_PRGF
**IFN** **α**	102,640 ± 39,659	126,643 ± 76,385	89,922 ± 49,238
**IL21**	39,370 ± 14,831	55,602 ± 36,939	51,241 ± 35,727
**TGF** **β** **1**	42,759 ± 57,933	41,074 ± 64,141	51,304 ± 68,296
**IL18**	22,678 ± 11,798	29,334 ± 17,542	23,004 ± 14,549
**IFN** **β**	22,621 ± 2,824	30,278 ± 38,887	17,901 ± 10,711
**NCAM1**	19,429 ± 1,683	19,607 ± 2,187	19,007 ± 863
**TIMP2**	11,865 ± 2,076	12,651 ± 2,442	12,227 ± 2,198
**IL1RA**	15,753 ± 6,046	10,051 ± 4,136	9287 ± 3,882
**IL17F**	11,133 ± 15,288	11,775 ± 18,228	10,160 ± 12,273
**IL12**	9,748 ± 4,021	7,539 ± 2,711	6,922 ± 2,083
**IL22**	9,826 ± 10,755	8,173 ± 9,469	5,632 ± 7,697
**TWEAKR**	7,387 ± 2,289	7,175 ± 2,629	7,586 ± 3,844
**OPG**	3,363 ± 4,232	3,981 ± 5,885	3,311 ± 4,041
**IL4**	3,046 ± 4,503	2,168 ± 2,821	3,293 ± 5,216
**EPO**	2,845 ± 1,273	2,989 ± 1,911	2,384 ± 1,141
**ANG1**	1,998 ± 1,722	2,283 ± 1,770	2,105 ± 1,405
**PDGFBB**	1,855 ± 367	1,948 ± 315	1,876 ± 349
**PlGF2**	1,030 ± 687	1,000 ± 420	1,244 ± 1,619
**IL13**	825 ± 1,000	921 ± 1,331	769 ± 834
**CCL3L1**	653 ± 419	853 ± 666	726 ± 426
**MIG**	330 ± 162	403 ± 251	318 ± 199
**MIP1B**	264 ± 177	341 ± 261	284 ± 208
**IGFBP5**	269 ± 343	298 ± 406	161 ± 174
**IL1** **β**	172 ± 50	260 ± 79	252 ± 134
**GMCSF**	207 ± 74	190 ± 75	181 ± 68
**TIMP1**	126 ± 42	290 ± 375	156 ± 100
**IL6**	228 ± 385	98 ± 110	157 ± 172
**DECORIN**	125 ± 52	210 ± 160	145 ± 78
**IL1** **α**	135 ± 227	121 ± 164	133 ± 225
**PECAM1**	108 ± 81	104 ± 58	92 ± 77
**TGF** **α**	101 ± 101	86 ± 85	64 ± 69
**IL17A**	67 ± 56	81 ± 68	57 ± 77
**MCP1**	41 ± 24	40 ± 21	35 ± 18
**TNF** **α**	18 ± 11	14 ± 11	15 ± 13

Mean values ± SD, n = 6.

**Table 3:** Correlation for soluble factors in P2_PRGF activated with 13.3 mM CaCl_2_.

(A)	IFNβ	TWEAKR	OPG	CCL3L1	MIG	MIP1B	IGFBP5	TIMP1	IL6	DECORIN	IL1α	PECAM1	IL17A	MCP1	TNFα
**IFN** **α**	0.829			0.943											
**TGF** **β** **1**						0.829								0.829	
**IL18**		0.943											0.829		
**TIMP2**									0.886			0.829			*−1.000*
**IL17F**			0.886							0.829	0.943				
**IL22**			0.943				0.943	0.943							
**OPG**							0.886		0.829	0.829					
**IL4**					0.943	0.943					0.829			0.943	
**EPO**						*−0.829*								*−0.829*	
**MIG**														0.886	
**MIP1B**														1.000	
**IGFBP5**								0.886					0.829		
**DECORIN**												0.943			*−0.886*
**(B)**	**PDGFBB**	**TWEAKR**	**IL17A**	**TGF** **β** **1**	**TIMP1**	**CCL3L1**	**TGF** **α**	**IFN** **α**	**IFN** **β**						
**PLT**	0.829	*−0.257*	*−0.371*	*−0.371*	*−0.429*	*−0.771*	*−0.771*	*−0.829*	*−1.000*						

Spearman r values ≥ 0.8 or ≤−0.8 with *p* ≤ 0.05 are shown in panel (**A**). Spearman’s r values with *p* ≤ 0.05 are shown in panel (**B**). Negative values in italics.

## Data Availability

The original data presented in the study are openly available at https://osf.io/5syfq/overview?view_only=13277e6957974405a60c499c3619ad20 (accessed on 22 December 2025).

## Supplementary Materials

The following supporting information can be downloaded at: https://www.mdpi.com/article/10.3390/biomedicines14030640/s1, Table S1: Quantification of soluble factors (pg/mL) in porcine versus human activated PRGF obtained with the identical device and protocol (P2) and analyzed with the identical ELISA platform in its porcine or human release [25].

## Abbreviations

The following abbreviations are used in this manuscript:

PRP	Platelet-rich plasma
PRGF	Plasma rich in growth factors
WB	Whole Blood
WBC	White blood cells
PLT	Platelet
RBC	Red blood cells
HGB	Hemoglobin
HCT	Hematocrit
MCV	Mean Corpuscular Volume
MCH	Mean Corpuscular Hemoglobin
MCHC	Mean Corpuscular Hemoglobin Concentration
NEUT	Neutrophils
LYMPH	Lymphocytes
MONO	Monocytes
EOS	Eosinophils
BASO	Basophils
ELISA	Enzyme-linked immunosorbent assay
CaCl_2_	Calcium chloride
